# You aren’t going to like what comes after America: Europe and global health after 2025

**DOI:** 10.1093/eurpub/ckag061

**Published:** 2026-06-11

**Authors:** Scott L Greer

**Affiliations:** School of Public Health, University of Michigan-Ann Arbor, Michigan, USA; European Observatory on Health Systems and Policies, Brussels, Belgium; Institute of Politics and Social Policy, Johannes Kepler University Linz, Austria

## The wood chipper

On 3 February 2025, less than 2 weeks into the second Trump administration, Trump’s centibillionaire lieutenant Elon Musk posted on his social media site that “we spent the weekend feeding USAID into the wood chipper. Could gone to some great parties. Did that instead [*sic*].” To the shock of those accustomed to the politically gridlocked and legally constrained American federal government, Musk and collaborators had done exactly what he said. Within days billions of dollars of expenditures were frozen, and the over ten thousand employees of the United States Agency for International Development (USAID) were recalled to the US and laid off (litigation about their firings continues).

The destruction of USAID was part of a year spent undoing what had been the United States’ global health policy. Across the new administration, actions ranged from withdrawing from the WHO and around eighty other international organizations to stopping payment on contracts to forcing out non-US-based members of research teams receiving US research grants.

**Figure 1.**
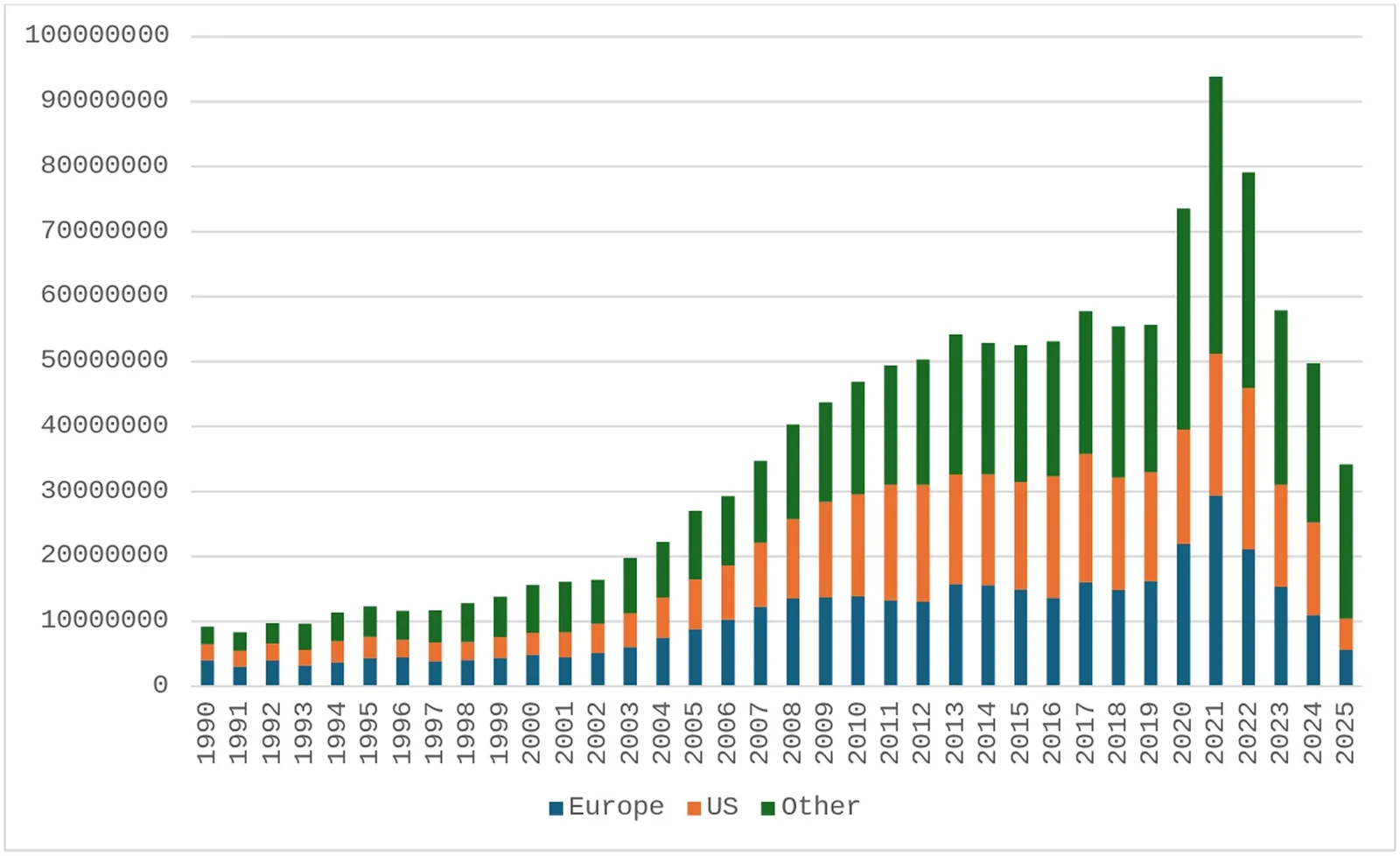
Development Assistance for health, 1990-2025, millions of 2023 US$. Source: IHME Development Assistance for Health Database (GHDx), 1990-2025. Europe refers to EU member states, Norway, Switzerland, and the UK. 2025 results are provisional and in Europe only refer to aid from France, Germany, the Netherlands, Norway, Spain, and the UK. http://vizhub.healthdata.org/fgh/, accessed 4 February 2026.

The results are dramatic (Fig. 1). The WHO lost around sixteen percent of its budget, more or less overnight. Billions of dollars of aid were subject to “rescissions” in which promised payments are not made. US health aid dropped from a COVID-19 peak of $23.3bn to $4.82bn. Parts of the PEPFAR, the program that pays for highly active anti-retroviral therapy against HIV in Africa, limp along, though what parts and with what resources remains obscure. Organizations, associations, and government ministries that relied on US aid for core or project funding are weakened, and people or groups that relied on implicit US protection are dangerously exposed. Decades of argument about whether US global health policy was morally or practically justified are moot, for that policy is gone.

The domestic health politics of the United States are also likely to spill over. In particular, Health and Human Services Secretary Robert F. Kennedy Jr. and his team, pursuing what they call the “Make America Healthy Again” agenda, have turned the force of the US federal government against science-based vaccine research and vaccination promotion. Policymakers in Europe, the world’s most vaccine-hesitant continent even before COVID-19, should expect to see increased resources, prestige, and momentum for vaccine opponents in their countries. The economics of vaccines will also change, as the US reduces funding for promising research (notably mRNA technology), directs funds into politically favored work, and becomes a less attractive market for vaccine developers. The astonishing fact that the US federal government is now encouraging research into measles treatments—having apparently decided to quit preventing measles—is small recompense.

## “America first”

The Trump/Musk cuts to USAID were made on the broad theory that the new administration should destroy programs it deemed suspicious and figure out later what might replace them. The “America First Health Strategy,” driven by the State Department, is an effort to replace the destroyed US global health strategy with something else. That something else is a series of Memoranda of Understanding with African states. The contents of these memoranda are opaque, but it appears that they offer the promise of restored funding in return for US private firms’ access to data and African compliance with other priorities of the Trump administration such as mineral rights or compliance with its “third country deportation” policies [1].

In the transit from the wood chipper to “America first,” we can see an effort to shift from destroying an imperfect but law-governed global health strategy into an approach in which global health is just part of the White House’s narrowly focused, transactional approach to affairs.

This centralized, personalistic approach has produced wildly unpredictable overall policy so far, with the US President levying high and confusing tariffs, threatening to annex neighboring allies, kidnapping the President of Venezuela, and bombing a wide range of countries in his first year. Any global health policy will lack coherence and priority in such a political system.

## A European opportunity?

One of the more optimistic takes—perhaps an optimism born of a lack of alternatives—is that the abrupt departure of the US from the global health order will allow others to replace it and perhaps improve it. Part of this is likely to mean greater self-reliance and cooperation among lower- and middle-income countries. But it is also an opportunity for Europe. In WHO, for example, the large European budget contributions and the large number of EU states and allies, it would be hard for Europeans to avoid becoming the dominant bloc in a post-American WHO.

Unfortunately, European governments’ budgetary constraints and domestic politics have not led them to adopt a position of global health leadership. In terms of direct health ODA, they mostly cut, hard, in 2025. Figure 1 shows declining global health aid, a decline that was clearly accelerating in 2025 [2]. Models suggest this declining aid will lead to enormous and inequitable preventable morbidity and mortality [3].

The EU’s budget formulation process runs on seven-year cycles, with the next seven-year framework to be settled in 2026. It remains to be seen what the EU’s contribution and priorities will be going forward, but if European states’ turn from global health is any sign, advocates of a prominent EU role in global health assistance have a difficult task.

The EU’s global health policy priorities are formally presented in its 2022 Global Health Strategy, a joint production of the DG for health (SANTE) and international aid (INTPA) that was ratified in 2024 Council Conclusions. The document is complex, containing many strong policy tools and statements, including a commitment to multilateralism and values such as gender equality.

But look at it in the context of the EU as a whole, not just SANTE and INTPA. Powerful EU policy instruments, especially trade, are not discussed in the Strategy. In the areas beyond the spotlight of the strategy, we can see another, less explicit, strategy. In this second strategy, the goal is strategic autonomy. That means urgently reshoring medical supply chains so that the EU is not dependent on fickle third countries and defending intellectual property, fully protected, in the hands of European companies [4].

## Rebuilding global health

As the Canadian artist Leonard Cohen wrote in 2003, “You aren’t going to like/what comes after/America.” It would be literally impossible to put back the US global health policy, or global politics, of 19 January 2025. The Trump replacement is a set of narrow and extractive bilateral memoranda that hardly amount to a policy. Americans will, at some point, have a chance to debate and create a global health policy worthy of whatever their country becomes.

In the meantime, European and allied leaders of rich democracies, worried about broader geopolitics and budgets, are declining to take up the mantle of global health leadership. Simple arithmetic says the EU and its member states, if they coordinate, are leading, if not the leading global health governance actors. But so far, they are cutting ODA, while focusing on supporting and reshoring their pharmaceutical industry. It is no surprise that the attention of policymakers in the rest of the world is turning to ways they can develop collaboration and greater self-reliance.

Governments, advocates, and researchers in high-income countries could do lots more [5]. The question is whether they will do it. Perhaps everybody needs to rethink global health policy to make it worthy of their country.
